# Pamphlets Received

**Published:** 1883-08-20

**Authors:** 


### *PAMPHLETS RECEIVED.*

Report of the Proceedings of the Illinois State Board of Health Quarterly Meeting, June 29tn, 1883.

State Control of Railroad Rates, Transportation Problems, etc, California.

Report on Diseases of Women, from the First Congressional District, by R J Nunn, M D, Savannah, Ga. [Reprint from the Transactions of the Medical Association of Georgia.]

Fifth Annual Announcement of The Southern Medical College, Atlanta, Ga. W P Nicolson, Dean.

One Hundred and Eighteenth Annual Announcement of the University of Pennsylvania Medical Department. James Tyson, M D, Secretary.

A Contribution to the Study of Neglected Lacerations or the Cervix Uteri and Perineum. by Thomas A Ashby, M D, Professor of Obstetrics, Woman's Medical College of Baltimore, etc, etc. [Read before the Clinical Society of Maryland, May 4th, 1883.

Credit: Its Meaning and Moment. By Clark W. Bryan, editor and proprietor of The Paper World, and Manufacturer and Industrial Gazette.

Antisepsis in Ovariotomy and Battey's Operation: Eighteen Consecutive Cases, All Successful. Battey.

